# Caffeic acid-integrated biopolymer systems: Advancing sustainable active packaging for food preservation

**DOI:** 10.1016/j.fochx.2025.102763

**Published:** 2025-07-14

**Authors:** Wanying Sun, Yuxiao Hu, Ananthi Pandi, Guohui Yi, Zhijian Tan

**Affiliations:** a Public Research Laboratory, Hainan Medical University, Haikou 571199, China; b Institute of Bast Fiber Crops & Center of Southern Economic Crops, Chinese Academy of Agricultural Sciences, Changsha 410205, China; c Department of Chemistry, The Gandhigram Rural Institute – Deemed to be University, Gandhigram, Dindigul 624302. Tamil Nadu, India

**Keywords:** Active packaging, Antioxidant, Film properties, Shelf life

## Abstract

The urgent need for sustainable food packaging solutions has propelled research into biopolymer-based materials enhanced with natural phenolic compounds. Caffeic acid (CA), a plant-derived polyphenol, stands out as a multifunctional modifier capable of addressing key limitations of biopolymers, such as mechanical fragility, moisture sensitivity, and insufficient antimicrobial activity. This review comprehensively explores CA's dual capacity to stabilize colloids and crosslink polymer chains in polysaccharide- and protein-based films, alongside advanced strategies like enzymatic grafting and nanocomposite design to optimize structural integrity, barrier performance, and active functionalities such as UV-blocking and pH-responsive release. Applications in preserving perishable foods—including meats, fruits, and seafood—demonstrate that CA-functionalized films significantly enhance mechanical strength, moisture resistance, and oxidative stability while delivering robust antimicrobial and antioxidant effects. Although covalent grafting outperforms physical blending in sustaining efficacy, challenges persist in thermal stability and scalable manufacturing. Active food packaging combined with CA will focus on release control optimization, material compatibility improvement, cost reduction and regulatory improvement in the future, to promote the expansion of natural and safe preservation technology to multiple food categories.

## Highlights

• CA stabilizes colloidal networks, enhancing film strength and barrier properties.
• CA-engineered hydrocolloids suppress lipid oxidation and microbial growth in food.
• Enzymatic grafting offers eco-friendly routes for CA integration into biopolymers.
• CA-composites enable UV-blocking, pH-responsive, and moisture-resistant packaging.

## 1 Introduction

In recent years, growing global awareness of environmental sustainability has accelerated research into biodegradable and functional packaging solutions, particularly within the food sector (Guo et al., n.d.-a, n.d.-b; Swarupa & Thareja, 2024). Among these innovations, biopolymer-based films and coatings have garnered significant attention due to their renewability, biodegradability, and compatibility with food products (He et al., 2025). Commonly used biopolymers such as chitosan, gelatin, starch, alginate, pectin, and polylactic acid (PLA) are favored for their excellent safety profiles and strong film-forming abilities. Nevertheless, despite their promising attributes, conventional biopolymeric materials are often hindered by functional drawbacks, including low mechanical strength, high water sensitivity, and limited antimicrobial and antioxidant properties (Devi et al., 2024; Su et al., 2025). To address these limitations, recent research has increasingly focused on incorporating natural bioactive compounds into biopolymer matrices to enhance their functional and protective properties (Cui et al., 2023). Among the natural bioactive compounds, caffeic acid (CA) has emerged as a promising candidate for functionalizing food packaging films. CA is a naturally occurring hydroxycinnamic acid, abundant in fruits, vegetables, coffee, and propolis (Dai et al., 2017; Rivera et al., 2024). Its phenolic structure imparts strong antioxidant and antimicrobial properties, enabling radical scavenging, metal ion chelation, and inhibition of lipid oxidation and microbial growth (Basumatary et al., 2022). In addition, CA exhibits UV-blocking potential and can interact with biopolymer chains through hydrogen bonding, π–π stacking, or covalent bonding, leading to improved film structure and stability (Thongphichai et al., 2023; F. Xie, 2023). Incorporating CA into biopolymer matrices not only enhances oxidative stability and antimicrobial defense but also improves mechanical strength and water resistance (Y. Wang, Du, et al., 2019). Furthermore, CA-functionalized films have been combined with nanotechnology approaches, such as metallic or metal oxide nanoparticles, to develop nanocomposites exhibiting synergistic antioxidant and antimicrobial effects (Yuan et al., 2023). Its participation in polymer crosslinking reactions enhances film integrity and enables controlled release of active agents, which is crucial for extending the shelf life of perishable foods (Benbettaieb et al., 2018; Lyu et al., 2024). Moreover, CA's ability to block UV radiation makes it ideal for protecting light-sensitive food products like oils, dairy, and beverages (Kaczmarek-Szczepańska et al., 2023; Ordoñez et al., 2022). Its environmental responsiveness—changing properties in reaction to pH, light, or microbial activity—also paves the way for the development of smart packaging materials that can monitor food freshness and safety (Khan et al., 2021; Luzi et al., 2020).

Despite its multifunctional potential, several technical challenges must be addressed before CA can be widely adopted in commercial food packaging applications (Lin et al., 2023). Key issues include its thermal instability, potential incompatibility with hydrophobic polymers, and difficulties in achieving uniform dispersion within polymer matrices (Pei et al., 2022). These limitations can compromise the stability and performance of CA-functionalized films during processing and storage. To overcome these barriers, various strategies such as encapsulation, emulsification, and chemical modification have been actively explored to enhance the stability, controlled release, and integration of CA into packaging systems (Bai et al., 2023). From a safety and regulatory perspective, CA holds an advantage as it is classified as a Generally Recognized as Safe (GRAS) compound, supporting its application in edible coatings and food-contact materials. Moreover, the growing consumer demand for clean-label, natural, and environmentally friendly packaging solutions further underscores the relevance of incorporating natural additives like CA into modern food packaging technologies. These trends align well with industry and regulatory movements toward reducing synthetic additives and promoting sustainable practices in the food sector (Yuan et al., 2023). This review systematically summarizes the latest research progress on the incorporation of CA into biopolymer-based food packaging materials, focusing on its functional roles, challenges, and integration strategies. The innovative aspect of this work lies in its comprehensive evaluation of CA's multifunctionality, stability enhancement techniques, and its prospects in developing next-generation active and intelligent packaging systems. This timely synthesis of knowledge is intended to provide valuable insights for future research directions and practical applications in sustainable food packaging technologies.

## 2 Overview of CA

**Fig. 1:**
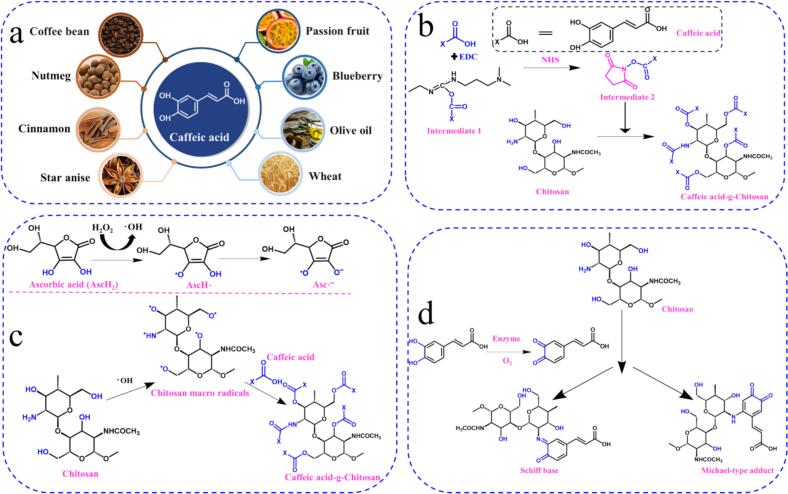
**(a)** Sources of CA. **(b)** Reaction mechanism of EDC/NHS chemical coupling for the synthesis of CA grafted chitosan. **(c)** Mechanism of ascorbic acid/H_2_O_2_ redox pair mediating the synthesis of CA-grafted chitosan by grafting. **(d)** Reaction mechanism of enzyme catalyzed grafting approach. Adapted from reported study (Liu et al., 2017a, 2017b).

CA (i.e., 3,4-dihydroxycinnamic acid) is a major phenolic compound broadly found as a secondary metabolite in a multitude of plants (Fig. 1a) such as nutmeg, cinnamon, star anise, wine, blueberries, coffee, wheat, olive oil, and beans (Khan et al., 2021). It has been reported that it is one of the most abundant phenolic acids in fruits, accounting for 75 to 100 % of the total hydroxycinnamic acid content (Pavlíková, 2023). Currently, CA is obtained by three main methods: solvent extraction (Li et al., 2021), chemical synthesis (Flourat et al., 2021) and biological fermentation (Arancibia-Díaz et al., 2023). Among them, there are many extraction methods for CA, such as water extraction, reflux extraction, ultrasonic extraction, microwave-assisted extraction, enzymatic method, enzymatic hydrolysis-ultrasonic combination method, supercritical extraction, etc. Chemical synthesis of CA usually uses o-hydroxybenzaldehyde (3,4dihydroxybenzaldehyde) and acrylic acid as the main starting materials. Then esterification, aldol condensation and decarboxylation are carried out to obtain CA (Sidoryk et al., 2018). The price range of CA sold in the market is 450–900 $/kg. Therefore, it exhibits a good economic worth. However, the extraction and purification of CA can be both complex and expensive, and the efficiency of the extraction method and purification process can affect the yield and quality of CA. Therefore, the development of efficient and sustainable extraction technologies is essential to ensure a continuous supply of high-quality CA.

CA is an organic acid, chemical formula for C_9_H_8_O_4_ (molecular weight: 180.15, density: 1.2933), yellow crystalline powder, skeleton for the C6-C3 structure, and contains two phenolic hydroxyl groups (Fig. 1a). Due to its chemical structure characterized by dihydroxyl and carboxyl functional groups, it exhibits significant antioxidant and antimicrobial properties. Furthermore, it also offers a variety of bio-activities such as anti-cancer and anti-inflammatory (Waheed et al., 2019), and is an important precursor for the synthesis of plant-derived aromatic chemicals such as rosmarinic acid, chlorogenic acid and phenethyl caffeate. Among them, the assessment of antioxidant capacity needs to be about thermodynamics (redox potential) and kinetic rate constants of different types of free radicals, stability and stoichiometric properties of antioxidant derived free radicals (Costa et al., 2020). The structure of CA is an effective structure for scavenging free radicals. The antioxidant behavior is due to the extended conjugated side chain of the aromatic ring allowing easy delocalization of unpaired electrons (Pavlíková, 2023). One way by which CA acts as an antioxidant is to quench free radicals by providing hydrogen atoms. o-Dihydroxy structures are natural free radical sites that produce o-semiquinone upon one-electron donation. One way in which CA serves as an antioxidant is by providing hydrogen atoms to quench free radicals. The o-dihydroxyl structure is a natural free radical site, yielding o-semiquinone upon one-electron donation. The lateral double bond conjugated to the o-hydroquinone moiety results in a wide range of electron delocalizations, increasing the stability and antioxidant activity of the o-semiquinone moiety. Another way in which CA acts as an antioxidant is by chelating metals through its two hydroxyl groups, and the o-hemiquinone moiety also serves as a preferred binding site for trace metals, leading to significant chelating activity (Marković & Tošović, 2016). Spagnol et al. demonstrated by in vitro modeling that CA, as an excellent antioxidant (including H_2_O_2_ trapping, ABTS trapping capacity and DPPH radical scavenging capacity), has significantly stronger antioxidant activity than ascorbic acid (Spagnol et al., 2019). Currently, the generally accepted antibacterial mechanisms of CA include: (1) reacting with the cell membrane, resulting in increased permeability and loss of cellular components; (2) inactivating enzyme systems or essential enzymes, affecting the production of energy and the synthesis of structural components required for bacterial growth; and (3) destroying the functional genetic material of bacteria (Zanetti et al., 2018). CA belongs to the group of phenylpropanes, which can inhibit the growth of *Escherichia coli*, *Staphylococcus aureus*, *Bacillus cereus*, *Listeria monocytogenes*, and some yeasts (Zhang, Roy, Assadpour, et al., 2023). CA can interact with bacterial multi-subunit proteins through ionic and hydrogen bonds to achieve bacteriostatic activity. It was found that CA at a concentration of 100–500 μg/mL could effectively inhibit the growth and reproduction of *Bacillus cereus*, *Listeria monocytogenes*, *Escherichia coli* and *Staphylococcus aureus*. The antimicrobial activity of CA is not only dependent on its structure, but also on environmental conditions. It has been demonstrated that the antimicrobial activity of CA is enhanced by simple acid-base complexation with spermine/spermidine (Mude et al., 2022). CA scavenges oxygen free radicals released by neutrophils and macrophages during the inflammatory response and significantly ameliorates leukopenia, oxidative stress and inflammation. It has been reported that CA inhibits LPS-induced damage to primary bovine mammary epithelial cells by inhibiting the activation of NF-κB and MAPK, and prevents apoptosis by inhibiting the activation of NF-κB and decreasing the expression of pro-inflammatory cytokines (Liu et al., 2019). Furthermore, the structure of CA contains a reactive carboxylate structure that can be amidated with amino groups and covalently grafted, thus improving its application in food products. For example, the grafting of CA onto chitosan involves three commonly used methods, including chemical coupling grafting, free radical-mediated grafting, and enzyme-catalyzed grafting [Figs. 1**(b-d)]**. The chemical coupling grafting method mainly uses 1-(3-Dimethylaminopropyl)-3-ethylcarbodiimide (EDC) and N-Hydroxysuccinimide (NHS) as crosslinking agents to form chitosan-CA graft copolymers by chemical reaction between the amino and hydroxyl groups of chitosan. EDC first reacts with the carboxyl group in CA to form o-acylisourea (intermediate 1). If the intermediate does not react with the amine, it will hydrolyze and regenerate the carboxyl group. When NHS is present in the system, the o-acylisourea will further react with NHS to form the active ester (intermediate 2). Finally, the reactive ester reacts with the amino or hydroxyl group on chitosan to form a chitosan-CA graft copolymer (Liu et al., 2016). Ascorbic acid can react with hydrogen peroxide in CH_3_COOH solution to form a hydroxyl radical (^•^OH) and a resonance-stabilized ascorbic acid tricarbonyl radical (AscH^•^), which exhibits a pK of −0.86, is not protonated and exists in the form of semi-dehydroascorbic acid radical (AscH^•―^). Subsequently, the forming hydroxyl radicals can capture hydrogen atoms from the chitosan molecule to form chitosan macromolecular radicals. Eventually, the CA monomer close to the reaction site serves as an acceptor for the chitosan macromolecular radicals, resulting in the synthesis of chitosan-CA graft copolymers (Liu et al., 2017; Suginta et al., 2013). Recently, enzyme-catalyzed grafting reaction has been gradually employed in the preparation of chitosan-CA graft copolymers. Compared with chemical coupling, enzyme-catalyzed grafting is safer and more environmentally friendly (Aljawish et al., 2014). Usually, laccase (EC 1.10.3.2), peroxidase (EC 1.11.1.x) and tyrosinase (EC 1.14.18.1) are the most commonly used enzymes to catalyze the synthesis of chitosan-CA graft copolymers (Yang et al., 2016). These enzymes can oxidize CA into quinone intermediates; quinone intermediates are highly active and electrophilic, and can further participate in a variety of non-enzymatic reactions, mainly through covalent coupling reactions such as C—C, C—O and C—N bonds, forming dimers, oligomers and polymers (Liu et al., 2017; Yang et al., 2016). Meanwhile, quinone intermediates can also react with chitosan in two different types of reactions, mainly through covalent bonds to produce Schiff base (C=N) or Michael addition reactants (C-NH). The graft modification optimized the insoluble properties of chitosan, meanwhile, it also endowed chitosan with new functional properties.

CA in the global food, pHarmaceuticals, and cosmetics market size was valued at approximately USD 140 million in 2022 and is expected to grow at a CAGR of more than 3.4 % from 2023 to 2032, due to the demand for CA driven by the growing consumer awareness of the health benefits of natural ingredients and the desire for natural and functional ingredients. The demand for CA in the food industry is primarily driven by the need for natural and safe ingredients to meet consumer preferences for cleaner labeling and healthier food choices. The main applications of CA in the food industry are as a functional agent for freshness preservation, a nutritional enhancer and a flavor modifier. Among them, it is particularly favored in the field of food preservation due to its excellent antimicrobial and antioxidant properties. For example, Fu et al. prepared agar-CA graft copolymer (Cf—Ag) by grafting CA onto agar. The DPPH radical scavenging rate was 50.09 % and Cf—Ag had excellent antimicrobial properties, with an inhibition rate of up to 100 % against *Escherichia coli* and *Staphylococcus aureus*. The results of applying Cf—Ag to fish preservation showed that Cf—Ag inhibited the bacterial growth and fat oxidation of fish during cold storage, and reduced the moisture loss, thus extending the overall quality of fish (Fu et al., 2023). Zhu et al. directly doped CA into poly (lactic acid) (PLA) solution to prepare activated nanofiber films by electrospinning technique. With the doping of CA, the films had enhanced water vapor barrier and surface hydrophobicity, and exhibited excellent antioxidant properties and storage stability. Eventually, the results of pork preservation application experiments showed that the active film effectively retarded the lipid oxidation and protein degradation of pork and maintained the meat color (Zhu et al., 2025). Furthermore, CA was also used for color enhancement and anthocyanin stability (Qian et al., 2017). Specifically, the cochromant enhances the color intensity and protects the colored scutellar cation from nucleophilic attack by water molecules. Based on this mechanism, freshness-indicating films with enhanced stability were prepared in the field of intelligent food packaging. He et al. prepared a color-enhanced smart film for visual monitoring of freshness using polyacrylonitrile (PAN) as a film-forming solution, blueberry anthocyanin as an indicator, and CA as an co-pigment (He et al., 2023). Shi et al. used CA (0–30 %, *w*/w) as a biobased modifier for interfacial interaction with Laponite nanosheets to prepare collagen composite films by solution casting method using collagen as the substrate. The composite films had enhanced hydrophobicity and barrier properties against water vapor. Finally, it was successfully applied to the real-time monitoring of shrimp freshness (Shi et al., 2024). Overall, CA has a good promising application in the food industry.

CAs have a broad range of pharmacological activities, including antioxidant, anticancer, antiviral and neuroprotective effects. This is due to their capabilities to inhibit free radicals, suppress the generation of pro-inflammatory cytokines, and modulate cell signaling pathways (Ehtiati et al., 2023). Although CA has been reported to have antimicrobial, anticancer and anti-inflammatory activities, it is still classified by the International Agency for Research on Cancer (IARC) as a potential human carcinogen in group 2B. This is mainly attributed to CA/copper-mediated DNA destruction, gene mutations and chromosomal aberrations. However, Li et al. demonstrated that chlorogenic acid (CGA) selectively regulates CA oxidation according to the concentration of copper ions in vivo. Moreover, CGA reduces the potential carcinogenic activity of CA and also promotes the autoxidation of CA to a level conducive to disease prevention, thereby making it an indispensable companion of CA (Li et al., 2023). Croft et al. indicated that CA has been reported to have health-promoting effects due to CA-quinone following CA autoxidation (Croft et al., 2017). Furthermore, the results of animal experiments show that CA in animal feed at a level of 0.05 % to 0.5 % can play an anticancer role (0.05 % is equivalent to 35 mg/kg). For regular coffee drinkers, the intake of CA is about 9 mg/kg per day. Even high coffee intake (10 mg/kg per day) is within the safe intake range of CA. Therefore, CA is a safe natural active substance. Additionally, Fırat et al. demonstrated that CA had a toxic effect on cancer cells, yet had little adverse effect on normal cells and did not decrease normal cell activity (Fırat et al., 2019). Currently, clinical trials are conducted on the safety of CA in humans (NCT 03070262). Despite its multiple active functions, more in vivo human studies are needed to further investigate the toxicity and safety of CA. The results of clinical trials will offer essential perspectives on its safety.

## 3 Direct addition of CA to functionalize biopolymer-based food packaging films

**Fig. 2:**
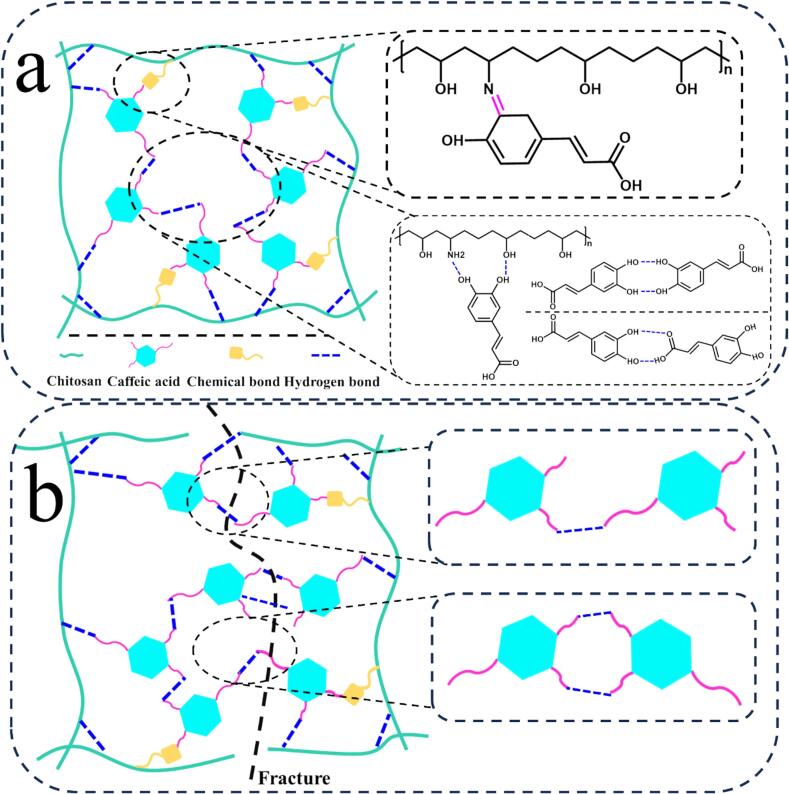
**(a)** The mechanism of interaction of CA directly doped into bio-based polymers (an example of chitosan). **(b)** Interaction of CA incorporation for mechanical strength increase. Adapted from reported study (Xu et al., 2022).

Nowadays, petroleum-based plastic packaging materials are ecologically harmful, so polysaccharides, proteins and other biopolymer materials are favored by a large number of researchers in the field of food active/smart packaging (Deng et al., 2025). These materials provide advantages over conventional plastic materials in terms of safety and eco-friendliness, including the ability to biodegrade, be recycled and non-toxic (Zhang & Rhim, 2022). Nevertheless, most natural materials lack the functional properties of antimicrobial and antioxidant properties.Therefore, different plant extracts are used as multifunctional additives to enhance the properties of biopolymer-based food packaging films, such as tannic acid and citric acid (Zhang, Roy, Assadpour, et al., 2023,Zhang, Roy, Ezati, et al., 2023). CA, a natural phenolic acid, has antimicrobial and antioxidant capabilities, good biocompatibility and relative safety, so it is often doped into biobased films to provide a variety of functional properties. In addition, the incorporation of CA into biopolymer-based films increases the mechanical strength of the films due to the covalent/non-covalent interactions between molecules (Fig. 2a). Antioxidant properties are usually characterized using free radical scavenging rates. Antimicrobial activity is characterized by inhibition of *Escherichia coli*, *Staphylococcus aureus* and fungi etc. Tensile strength and elongation at break are used to characterize mechanical properties. Doping into biopolymer-based films by direct approach has been focused on.

Polysaccharides are macromolecular compounds consisting of multiple monosaccharide molecules linked by glycosidic bonds, usually consisting of more than 10 monosaccharide units (Guo et al.). Polysaccharides are widely dispersed in nature and can be obtained from animals, plants and microorganisms, including cellulose, starch, chitosan, bacterial cellulose and so on. The naturalness, wide range of sources, biodegradability, good biocompatibility and excellent film-forming properties of polysaccharides have made them a commonly used substrate material for bio-based food packaging films (Yao et al., 2024). For example, Benbettaïeb et al. used chitosan and fish gelatin as the substrates for film formation and CA (5 %, *w*/w) as the functional component to prepare activated films by solution casting method. The findings revealed a significant decrease in the water vapor permeability (WVP) of the films with CA doping, suggesting that the doping of CA enhanced the barrier properties. Furthermore, the tensile strength (TS) of the CA-doped films increased by 35 %. These could be due to the fact that CA can interact with several active sites of fish gelatin and chitosan. For example, hydrogen-bonding interactions between CA and film-forming substrates; dimer-dipole interactions between phenolic hydroxyl groups in the CA structure and polar groups of proteins; and π-π stacking interactions between proteins and polyphenols can also be achieved via the nonpolar aromatic rings of polyphenols and aromatic amino acids (proline, phenylalanine, tyrosine, tryptophan, histidine). These interactions resulted in cross-linking of proteins and polysaccharide glycoside chains between them, leading to enhanced barrier and mechanical properties. The active film also exhibited excellent antioxidant activity (90 % DPPH radical scavenging) (Benbettaieb et al., 2018). Osondu et al. prepared an active coating by doping CA (0.07 %, *w*/w) directly into a chitosan hydrochloride (CH) substrate. The results of the study showed that CA incorporation reduced the occurrence of anthracnose in avocados during refrigeration and transportation, which could be attributed to the fact that CA activates the avocado's own defense mechanisms and may act as an adhesion agent between the pathogen and the avocado, thus facilitating CA-fungal interactions and cellular damage. Thus, the active coating holds promise as an alternative to conventional fungicides, providing a new strategy for preservation (Osondu et al., 2022). Szulc et al. prepared chitosan (CS)-CA active films by solution casting with CS and CA. The results showed that the Young's modulus of the films was enhanced by about 25 % and the elongation at break (EB) was reduced by 60 % after doping with CA. This may be attributed to the non-covalent interactions between chitosan and CA, such as hydroxyl and amino groups on chitosan molecules and phenolic hydroxyl and carboxyl groups on CA molecules, which promoted the intermolecular interaction forces of the films to become more compact (Fig. 2b). In addition, the CA-doped films have good thermal stability properties (Szulc & Lewandowska, 2023). Xu et al. prepared CA-containing active films (CCA) by solution casting method using chitosan as a film-forming substrate doped with CA (20 %, *w*/w) as a functional component. The results showed that the tensile strength (TS) and Young's modulus of the films increased to 58 MPa and 2783 MPa, respectively, with the doping of CA. this was attributed to the Schiff base cross-linking reaction between the amino group of chitosan and the o-quinone structure of CA to enhance the TS of the films. The CA-doped film had a DPPH radical scavenging rate of 95.62 %, an improvement of about 155 %, and enhanced UV-blocking properties. In addition, the water vapor barrier property and water resistance of the CA-doped films were enhanced. This was attributed to the formation of a chemical bonding network by the Schiff base reaction between CA and chitosan, and the internal structure became dense, making it more difficult for water vapor to pass through the films. However, the thermal stability of the films decreased. Although the Schiff base reaction between CA and chitosan improves the cross-linking degree, it affects the original hydrogen bonding, which has high thermal stability, thereby reducing the thermal stability (Xu et al., 2022). Fan et al. prepared electrospun fiber films by using electrospinning technique by homogeneously mixing functional components such as high straight chain corn starch (HACS) and CA (10 %, *w*/w). The results indicated that the CA-doped electrospun fiber films had high crystallinity because of the complexation between HACS and CA. In addition, the thermal stability of the electrospun fiber films with the addition of doped CA was improved (Fan et al., 2024). Yu et al. prepared active films with *N*, *O*-carboxymethyl chitosan (NOCC) and methylcellulose (MC) as the main film-forming substrates and CA (0.4 %, w/w) as the active ingredient by the solution casting methods. The results demonstrated that the DPPH and ABTS radical scavenging rate of the active films increased 20-fold and the bacteriostatic activity increased 6-fold with the doping of CA. Additionally, the film showed significant inhibition of lipid oxidation in herring oil-in-water emulsions (Yu et al., 2013). Kaczmarek-Szczepańska et al. mixed chitosan, CA (1 %, *w*/w), and polyethylene glycol and then prepared reactive films using a solution casting method. The findings indicated a decrease in the water vapor barrier properties of the films, due to the presence of hydrophilic groups in CA. CA can improve the mechanical properties by hydrogen bonding cross-linking with chitosan. The active film showed 93 % antioxidant activity. Furthermore, it showed excellent antimicrobial activity against *Escherichia coli* and *Staphylococcus aureus* (Kaczmarek-Szczepańska et al., 2021). Lin et al. used tapioca starch and sodium carboxymethylcellulose as film-forming substrates and CA nanosilica inclusion (1,5, w/w) as a functional component to prepare reactive films by solution casting method. The results showed that the CA-containing films had a good mechanical strength, and the tensile strength (TS) increased from 7.14 MPa to 15.44 MPa. The activated films showed good antioxidant activity, with scavenging rates of 95.43 % and 84.67 % for ABTS and DPPH radicals, respectively. The doping of CA also improved the thermal stability of the films. In addition, the inhibition rate of *Escherichia coli* and *Staphylococcus aureus* is 99.9 %, which can effectively maintain the quality of meat and prevent the microbial infection and oxidation of meat in the actual meat sample preservation experiment (Lin et al., 2023).

Currently, protein-based materials are categorized into two main groups: plant proteins (corn alkyd proteins, soybean isolate proteins) and animal proteins (chicken gelatin, fish gelatin). Therefore, they can be completely degraded in the natural environment after film formation, avoiding “white pollution”. Most of the protein materials are edible and can be used directly in food packaging with high safety. Furthermore, protein-based films are endowed with excellent film-forming properties and excellent air barrier function. However, the mechanical strength of protein-based materials is still relatively low and the antimicrobial and antioxidant activity is almost non-existent. Therefore, the combination of protein-based materials with CA, which has natural active effects and cross-linking effects, is of great interest. Many studies in this regard have also been reported in recent years. For example, Erge et al. prepared active films by solution casting method using chicken gelatin as the film-forming substrate and CA (1.25 %, *w*/w) as the active functional component. The results showed that the water vapor barrier property of the active films was enhanced with the incorporation of CA. This was due to the cross-linking that occurred in the active films which resulted in a tighter network of the film substrate, thus reducing the passage of water vapor through the film. The presence of CA increased the tensile strength (TS) of the active films. This was attributed to the increased intermolecular interactions and decreased free space between molecules by CA. In addition, the cross-linking effect exerted by CA doping improved the thermal stability of the films (Erge et al., 2024). Kang et al. prepared active films using soybean isolate protein (SPI) and CA (1.5 %, *w*/w) as the main film-forming substrate and active ingredient, respectively. The results showed that the TS of the active films was significantly enhanced by CA incorporation. The TS and Young's modulus of the pure SPI films were 3.1 and 123 MPa, respectively, whereas the TS and Young's modulus of the SPI-CA films were 6.5 and 233 MPa, respectively. These indicated that CA exerted a cross-linking effect in which it improved the interaction within the film and enhanced the mechanical strength. In addition, the incorporation of CA enhanced the water resistance and thermal stability of the active film (Kang et al., 2016). Nuthong et al. prepared active films by direct mixing of porcine plasma proteins and CA (1–3 %, w/w) using solution casting method. The results showed that the TS of the films increased significantly with the increase in CA incorporation. This could be due to the ability of CA to reacted with multiple protein sites and resulted in cross-linking between the proteins, thus enhancing the strength of intermolecular interactions of the films. This could be due to the ability of CA to react with multiple protein sites and resulted in cross-linking between the proteins, thus enhancing the strength of intermolecular interactions of the films. As CA was doped, the WVP of the active films increased from 2.80 × 10^−10^ g^−1^ s^−1^ Pa^−1^ to 2.99 × 10^−10^ g^−1^ s^−1^ Pa^−1^, which indicated a reduction in the water vapor barrier properties of the films. This may be due to excessive cross-linking of CA, leading to the formation of protein bundles in which water molecules can penetrate more easily (Nuthong et al., 2009). Guler et al. prepared active films using a centrifugal spinning strategy with gelatin as the base film-forming solution and CA (2–3 %, *w*/w) as the functional component. The findings showed that the incorporation of CA had no significant effect on the viscosity and fiber diameter of the gelatin solution. Moreover, the water vapor permeability (WVP) of the films increased with the doping of CA, which reduced the barrier properties of the films against water vapor. This may be due to the presence of polar groups in CA. The antioxidant properties of the films increased with the increase in CA content. Finally, the film was successfully applied to mitigate lipid oxidation of olive oil (Guler et al., 2024). Parsaei et al. prepared gelatin-based edible films by solution casting method by directly doping CA (1, 3 and 5 %, *w*/w) as a cross-linking agent and mixing it into fish gelatin film-forming solution. The results showed that the water solubility of the films decreased significantly with the doping of CA. Meanwhile, the WVP and oxygen permeability (OP) also decreased, which indicated that the barrier properties of the films were enhanced. With the increase of CA content, the TS and Young's modulus of the films increased and the mechanical properties were improved. These may be attributed to the cross-linking effect of CA, which enhanced the internal network interactions of the film, thus enhancing the compactness of the film (Parsaei et al., 2022).

A small number of studies have been reported on other biopolymers compared with polysaccharide- and protein-based materials. Ignatova et al. used a one-pot electrospinning strategy to dope CA (5, 10 and 20 %, *w*/w) into poly(3-hydroxybutyrate) (PHB) fibrous films, resulting in the preparation of active fibrous films. The findings demonstrated that the DPPH radical scavenging rate of the fiber film was enhanced from about 5 % to 75 % after CA doping. In addition, the CA-doped fibrous films showed excellent antimicrobial activity in bacteriostatic assays, with almost 100 % lethality against *Escherichia coli* and *Staphylococcus aureus* (Ignatova et al., 2016). Narayanan et al. doped CA cyclodextrin inclusion complexes (10 %, w/w) directly into polyvinyl alcohol (PVA) nanofibers by electrospinning technique. The results showed that the electrospun fiber films exhibited excellent inhibition of *Escherichia coli* and *Staphylococcus aureus*, which is promising for application in active packaging films (Narayanan et al., 2021).

## 4 CA grafted functionalized food packaging film

Chemical grafting of phenolic acids, particularly CA, onto biopolymer matrices has emerged as an effective strategy to enhance the mechanical, barrier, antioxidant, and antimicrobial properties of biodegradable films. Among the commonly employed grafting techniques, esterification, amidation, and radical-induced coupling reactions stand out for their ability to form stable covalent bonds between CA and polymer chains (Yu et al., 2013). Grafted films exhibit improved structural integrity, reduced water sensitivity, enhanced oxidative stability, and prolonged antimicrobial effectiveness compared to non-grafted counterparts. Recent studies have demonstrated that CA grafting not only preserves the inherent biodegradability of the biopolymer but also imparts additional active functionalities essential for modern food packaging applications (Zheng et al., 2023). Furthermore, optimized grafting conditions, such as pH control, catalyst selection, and reaction time, are critical to achieving uniform CA distribution and maximizing the functional benefits without compromising film transparency or flexibility. Ongoing research is focused on scalable grafting processes and the development of multifunctional films capable of intelligent response to environmental stimuli, paving the way for advanced active packaging systems. (Zhu et al., 2024). These reactions allowed for the covalent integration of CA molecules into the polymer backbones or side chains, enhancing the permanence of the active compound within the film (Kaczmarek-Szczepańska et al., 2021). For example, in gelatin and chitosan matrices, carbodiimide-mediated esterification enabled efficient binding of CA to amino and hydroxyl groups, forming stable grafted networks (Zhou, Han, et al., 2023). Radical-induced grafting using ascorbic acid and hydrogen peroxide as initiators also facilitated CA incorporation onto starch and cellulose backbones. In a study by (Fei et al., 2022), CA was grafted onto carboxymethyl cellulose films via EDC/NHS chemistry, and the grafting efficiency reached 78 %, indicating successful conjugation. Compared to physical adsorption, which showed less than 30 % retention under washing conditions, covalent grafting significantly improved the stability and persistence of CA in the film matrix (İlyasoğlu et al., 2019).

**Fig. 3:**
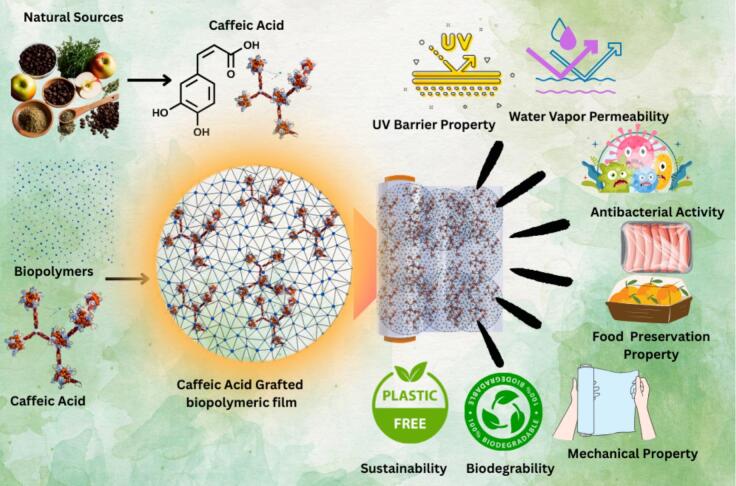
CA grafted functionalized food packaging films.

Advanced functionalization techniques such as enzymatic grafting using laccase or tyrosinase have also been explored to promote greener chemistry routes for CA grafting. These enzymes catalyze the oxidation of CA to quinones, which subsequently form covalent linkages with nucleophilic groups in biopolymers (İlyasoğlu & Guo, 2019). For instance, (Abdelmalek et al., 2023) demonstrated laccase-mediated CA grafting onto chitosan, which achieved 60 % higher antioxidant retention and 40 % increased grafting efficiency compared to chemical methods (Bai et al., 2023). Additionally, microwave-assisted and ultrasound-assisted grafting methods were developed to accelerate reaction kinetics, reduce solvent usage, and enhance energy efficiency (Saelo et al., 2016). These modern techniques were particularly advantageous for industrial scalability, offering time-saving and eco-friendly alternatives to conventional approaches. Hence, the method of grafting played a crucial role in determining the structural uniformity, efficiency, and functional efficacy of CA-modified biopolymer films (Araghi et al., 2015). The schematic diagram of CA grafted functionalized food packaging films as shown in Fig. 3**.**

The mechanical properties of biopolymer films, such as tensile strength (TS), elongation at break (EAB), and Young's modulus, were critically important for maintaining integrity during handling, transportation, and storage. Incorporation of CA through grafting significantly altered these mechanical attributes (Yong et al., 2021). The interaction between CA's phenolic hydroxyl groups and the polymer backbone enhanced the molecular interactions within the matrix, often resulting in denser and more cohesive networks (Zeren et al., 2022). In gelatin films grafted with CA, tensile strength increased from 23 MPa to 35 MPa, while the elongation at break slightly decreased, indicating a trade-off between strength and flexibility (Rivera et al., 2024). Several studies reported that CA grafting improved the rigidity and cohesion of the polymer chains due to hydrogen bonding and possible covalent crosslinking. For example, starch films functionalized with CA exhibited a 25 % increase in tensile strength and a 15 % reduction in water vapor permeability, suggesting tighter polymer packing (Rivera et al., 2025). Comparatively, the mechanical improvements were greater in grafted films than in those with physically incorporated CA, where the phenolic compound often acted as a plasticizer rather than a structural enhancer. Physical blends typically showed marginal TS increases or even reductions at higher CA concentrations due to phase separation or migration of free CA to the surface (Ounkaew et al., 2018).

In CA-grafted chitosan films, researchers observed an increase in Young's modulus, which reflected enhanced stiffness and structural resistance to deformation. This was attributed to additional crosslinking points provided by CA molecules, reinforcing the film matrix (Parsaei et al., 2022). Moreover, CA incorporation also reduced film thickness and porosity, both of which contributed to the improvement of mechanical performance. In comparison, enzymatic grafting methods offered slightly better results than chemical grafting due to more uniform distribution and localized bonding, which avoided brittleness (Arrua et al., 2010). Therefore, the mode of CA integration, the type of polymer matrix, and the grafting technique collectively influenced the mechanical profile of the films, with grafting proving superior in most reported findings (Guler et al., 2024).

The barrier properties of the films against water vapor and gases also underwent notable changes following CA grafting. The incorporation of CA, which possessed a hydrophobic aromatic ring structure, decreased the overall hydrophilicity of the films (Nunes et al., 2013). This led to a significant reduction in WVP, particularly in starch- and cellulose-based films, which were otherwise highly water-permeable (Huang et al., 2022). Additionally, the films' oxygen permeability was reduced due to denser polymer packing and reduced free volume in the film matrix. This densification hindered the diffusion of gas molecules through the film, thus enhancing the packaging's protective function, especially for oxygen-sensitive foods such as dairy and meat products. For instance, (Düz et al., 2024) reported a 40 % reduction in WVP for CA-grafted starch films, compared to only 18 % in films containing physically mixed CA. Similarly, gelatin-CA films showed a 30 % decrease in oxygen permeability, as opposed to a mere 12 % in gelatin films containing free CA. Thermogravimetric analysis (TGA) and differential scanning calorimetry (DSC) showed enhanced thermal stability in CA-grafted films (Xu et al., 2022). The degradation onset temperature shifted to higher values, and the total weight loss was reduced, suggesting increased resistance to thermal decomposition. This was particularly advantageous for packaging applications involving pasteurization, heat sealing, or hot filling. The improved thermal properties were a result of the strong interaction between CA and the polymer matrix, which restricted chain mobility and delayed thermal degradation. A comparative study by (Xu et al., 2022) indicated a 25 °C shift in degradation temperature in CA-grafted gelatin films, whereas a minimal 10 °C shift was observed in films with non-covalent CA incorporation. Similarly, CA-grafted cellulose films exhibited an increase in glass transition temperature by approximately 12 °C, indicating enhanced thermal resilience for industrial processing (Narayanan et al., 2020).

The incorporation of CA into biopolymer-based food packaging films had significantly enhanced their antioxidant properties due to the rich phenolic content of CA, which effectively donated hydrogen atoms to neutralize free radicals (Hazer et al., 2023). The presence of hydroxyl groups in CA facilitated radical scavenging mechanisms, which played a pivotal role in delaying oxidative degradation of both the packaging material and the packaged food. For example, CA-grafted chitosan films exhibited a remarkable increase in DPPH radical scavenging activity, from 42 % in neat chitosan films to over 85 % post-grafting, as reported by (Stojković et al., 2013). Similarly, starch-based films functionalized with CA showed a two-fold increase in ferric reducing antioxidant power (FRAP) compared to unmodified counterparts, illustrating the potent antioxidant contribution of grafted CA molecules.

Comparative studies consistently highlighted the superiority of CA-grafted films over those blended with free CA. While blending could initially enhance antioxidant capacity, the migration of CA over time reduced efficacy during prolonged storage (Olejnik & Masek, 2020). In contrast, grafted CA remained covalently bound, ensuring sustained antioxidant performance. In a notable example, (Sahraeian et al., 2024) observed that gelatin films with physically mixed CA lost nearly 50 % of their antioxidant activity after 14 days, whereas CA-grafted films retained over 90 % of their initial efficacy. Furthermore, films grafted enzymatically with CA using tyrosinase displayed prolonged oxidative stability under accelerated shelf-life conditions (Ma et al., 2024). These findings emphasized the long-term functional integrity of CA-grafted films and their advantage in preserving food freshness. In addition to antioxidant properties, CA-functionalized films demonstrated improved antimicrobial efficacy against foodborne pathogens such as *Escherichia coli*, *Staphylococcus aureus*, *Listeria monocytogenes*, and *Salmonella typhimurium* (Y. Wang, Xie, et al., 2019). The antimicrobial action of CA was attributed to its ability to disrupt microbial cell walls and inhibit enzyme activity essential for bacterial survival. For instance, CA-grafted gelatin films inhibited over 90 % of *E. coli* growth, as compared to 65 % by non-grafted films with free CA (Liu et al., 2024). Another study showed that chitosan-CA films exhibited a bactericidal effect within 4 h of contact, with significantly higher zones of inhibition than the physical blend. The synergy between CA and biopolymers was particularly notable in enhancing antimicrobial performance (İlyasoğlu et al., 2019). When CA was covalently grafted onto chitosan or alginate, the electrostatic interactions between the polymer and bacterial cell membranes were amplified, leading to more effective disruption (Jabłoński et al., 2022). Additionally, CA's phenolic structure facilitated ROS (Reactive Oxygen Species) generation, which further contributed to bacterial inactivation. The antimicrobial efficacy of such films was confirmed through in vivo studies as well. In fresh-cut fruit packaging trials, CA-functionalized films extended microbial shelf life by over 6 days compared to controls, while maintaining color and firmness (Jagadeesan et al., 2022). Hence, the dual functionality imparted by CA greatly improved the protective potential of biodegradable packaging films, offering active preservation without synthetic additives (Gangadharan et al., 2024).

Microscopic and spectroscopic analyses revealed considerable structural and morphological changes in the films post-grafting. SEM images of CA-functionalized films showed a smoother and more compact surface morphology compared to their pristine counterparts, which typically displayed surface cracks and pores (Klein & Poverenov, 2020). FTIR spectra confirmed the formation of new ester or amide bonds, indicating successful grafting of CA. XRD patterns showed decreased crystallinity in certain polymers, such as gelatin, due to disruption of the original ordered structure by CA incorporation (Gupta et al., 2025). Conversely, in starch films, a slight increase in crystallinity was observed, suggesting improved polymer chain alignment. AFM analysis revealed a significant decrease in surface roughness, which enhanced film transparency and made the films more suitable for high-end food packaging. Similar findings were reported by (George Kerry et al., 2018), who noted a 65 % reduction in RMS roughness in CA-grafted chitosan films. CA grafting modified the surface energy and hydrophilic/hydrophobic balance of the films. Contact angle measurements revealed that the surface of the grafted films became more hydrophobic (Nogueira et al., 2020). For instance, the contact angle of starch-based films increased from 56° to over 85° after CA grafting. This alteration in surface energy not only contributed to water resistance but also improved the films' performance in humid environments (Bajer, 2024). The more hydrophobic surface also reduced the likelihood of microbial colonization and biofilm formation, making the films safer for food contact applications. In comparative assessments with citric acid and tannic acid-modified films, CA-grafted films displayed superior balance between hydrophobicity and mechanical integrity (Pawłowska & Stepczyńska, 2022; Revutskaya et al., 2024). CA functionalization slightly altered the optical properties of films. While the films acquired a mild yellow or amber tint due to the presence of phenolic chromophores, they still maintained acceptable transparency in the visible range. UV–visible spectroscopy showed improved UV-blocking capacity, which protected light-sensitive foods from photodegradation (Ozuna-Valencia et al., 2024). The color stability of the grafted films was superior to that of physically incorporated films, as the covalently bound CA resisted photobleaching and oxidation under ambient light exposure. A study by Zhao et al. showed that the L* value of CA-grafted films remained stable over 30 days, whereas films with free CA experienced significant discoloration (Dai et al., 2017; Shen et al., 2023).

The functional performance of CA-modified films was extensively evaluated, demonstrating advantages over conventional synthetic packaging. Covalent grafting of CA onto biopolymers enabled controlled release of active compounds, overcoming limitations of physical blending (Singh et al., 2023). Migration studies confirmed minimal CA leaching, ensuring sustained functionality and safety (Sutil et al., 2022). Compared to non-grafted films, grafted systems retained CA more effectively, with 55 % less loss within 48 h. In multi-component blends, CA improved polymer compatibility, enhancing interfacial adhesion in gelatin-PVA and chitosan-alginate systems to yield uniform, mechanically stable films (Manzoor et al., 2022). For nanocomposites, CA facilitated nanoparticle dispersion (e.g., ZnO, AgNPs) by interacting with both polymers and nanofillers, amplifying antimicrobial and mechanical properties (Shi et al., 2024a). These advancements highlight CA's versatility in optimizing packaging performance while maintaining biodegradability. Although CA grafting slightly delayed the degradation rate of films due to increased matrix density and reduced hydrophilicity, the materials remained biodegradable. Soil burial and composting studies demonstrated that the grafted films underwent gradual enzymatic breakdown, releasing non-toxic byproducts into the environment (Wang, Liu, et al., 2021; Q. Xie et al., 2022). The presence of CA also enhanced the films' resistance to premature degradation during storage, increasing their usability without compromising sustainability. CA grafting significantly enhanced the physicochemical, structural, and functional properties of biodegradable food packaging films (S. Chen et al., 2024). Improvements in mechanical strength, barrier properties, antioxidant and antimicrobial activity, and thermal stability made these materials promising candidates for replacing synthetic packaging. Their biodegradable nature, minimal migration, and food-specific effectiveness further supported their commercial viability.

## 5 Application of CA functionalized food packaging films/coatings in food preservation

CA is extensively applied in food preservation due to its excellent antimicrobial, antioxidant and relative safety properties. This chapter examines recent advances in CA doping for the preparation of active food packaging films/coatings and evaluates their efficacy in preserving various food products (fruits and vegetables, meats, and seafood) (Table 1 **and** Table 2).

**Table 1:** Studies on CA-containing active films for various food preservation applications.

Package type	Preserved food	Significance	Reference
Active film	Mango and apple	Doping CA exhibits excellent DPPH free radical scavenging rate. CA-containing films provide better protection against fruit water loss and a slowing of fruit decay, as well as effective inhibition of *Escherichia coli* and *Staphylococcus aureus.*	(Xu et al., 2022)
Active film	Apricots (*Armeniaca vulgaris* Lam. Saimaiti)	CA-g-CS inhibited the growth of *Escherichia coli, Staphylococcus aureus and Bacillus subtilis.* It can better maintain the hardness, weight loss, SSC, TA, relative conductivity and respiration rate of apricot.	(Li et al., 2021)
Active film	Saimaiti apricots	CA-g-CS significantly reduced the weight loss and decay rate of ‘Saimaiti’ apricots, maintained high fruit firmness, soluble solids content and titratable acidity content, reduced the respiration frequency, effectively inhibited the increase of MDA content and relative conductivity, and inhibited the PPO activity, while enhancing the POD activity.	(Han, Sun, Song, & Chen, 2025)
Active film	Peach	CA-g-CS/Pul film is antifungal. It is effective in slowing down peach skin wrinkling, flesh collapse, browning, and mass growth of fungi that cause decay. Reducing the rate of spoilage, weight loss and MDA content. It could extend the shelf life of peaches by 2 days.	(Mou et al., 2025)
Active film	olive oil	Gelatin-based reactive fiber film led to a significant 45 % reduction in total oxidation of olive oil.	(Guler et al., 2024)
Active film	*Agaricus bisporus*	CA-g-CS/PLA film could reduce color, hardness and respiration rate changes. It decreased the concentration of carbon dioxide during storage, inhibited the accumulation of ROS, and ultimately delayed the browning and decay of *Agaricus bisporus*, and improved the shelf-life.	(Pei et al., 2022)
Active film	*Agaricus bisporus*	CA-g-CS/PLA retarded the increase in cell membrane permeability after harvesting of *Agaricus bisporus* and promoted proline accumulation to maintain cell morphology. CA-g-CS/PLA retarded the ROS activation of transcription factors upstream of membrane lipid degrading enzymes to inhibit expression.	(Zhou, Guo, et al., 2023)
Active film	*Agaricus bisporus*	CA-g-CS/PLA retarded the deterioration of postharvest volatile flavor of *Agaricus bisporus* by inhibiting the growth of dominant bacteria such as *Fusobacterium, Citrobacter, Hafnia-Obesumbacterium, Bacteroides, Pedobacter, Flavobacterium, Duganella and Pseudomonas.*	(Bai et al., 2023)
Active film	Pork	Adding CA has excellent antimicrobial and antioxidant properties. Less color difference, less odor, lower TVB-N and TBARS value than ordinary film-packed pork. Extends the shelf life of pork to 10 days at 4 °C.	(Yong et al., 2021)
Active film	Pork	CA-containing active fiber film preserved pork decreased the color change of meat, slowed down the increase of pH, TVB-N and MDA, and inhibited the growth and development of microorganisms.	(Zhu et al., 2025)
Active film	Beef and chicken	Meat products preserved with CA-containing active films exhibited better elasticity, firmness, stickiness and chewiness. Reduced color change of meat and slowed down the growth of pH and MDA. Restricted the growth and development of microorganisms.	(Lin et al., 2023)
Active film	*Penaeus vannamei*	CA-g-CS film effectively delayed the weight loss, pH rise, volatile alkaline nitrogen accumulations and microbial growth of *penaeus vannamei* during storage.	(Huang et al., 2022)

**Table 2:** Studies on CA-containing active coatings for various food preservation applications.

Package type	Preserved food	Significance	Reference
Active coating	Avocado	CH-CA coating exhibited antifungal activity. CH-CA coated preserved avocados had better overall acceptance, skin color, taste, flavor and texture structure.	(Osondu et al., 2022)
Active coating	Mulberry fruit	CTS-g-CA reduced the decay rate, MDA and weight loss. Enhanced free radical scavenging activity against DPPH and ABTS.	(Yang et al., 2016)
Active coating	Grass carp	The active coating doped with CA inhibited the growth of histamine-producing bacteria, lowered the rate of fat oxidation, reduced the production of free fatty acids, and significantly slowed down the increase in malondialdehyde (MDA) content of grass carp during storage. It slowed down the evaporation of water from the fillets, prolonged the shelf life of the fish and improved the overall quality of the fish.	(Fu et al., 2023)
Active coating	Pork	Cf-CTS coating provided the highest sensory score during storage. It can inhibit the growth of microorganisms and slow down the fat oxidation of pork.	(Fei et al., 2022)
Active coating	Pork	CA@CTS-E coating can inhibit microbial activity and antioxidant properties, reduce pH and MDA of pork during storage. It can extend the shelf life of pork by up to 6 days.	(Huang et al., 2022)
Active coating	Sausages	The CA-containing active coating significantly inhibited microbial growth and improved shelf life.	(Abdelmalek et al., 2023)
Active coating	Small yellow croaker	The CA-incorporated active coating inhibited the propagation and growth of microorganisms, slowed down the water loss rate of small yellow croaker fillets, and inhibited protein decomposition and fat oxidation.	(Wang, Fei, et al., 2021)
Active coating	Turbot	The active coating prepared with chitosan and CA retarded the increase in volatile saline nitrogen, colony counts and pH and reduced the decrease in water holding capacity in turbot.	(Tang et al., 2024)
Active coating	Pompano	CS-g-CA coating can effectively inhibit the growth and reproduction of total viable bacteria, *Pseudomonas*, *Cryptophilus* and H_2_S-producing bacteria. It reduced water evaporation, slowed down pH rise, better maintained the hardness and elasticity of pomfret, and reduced protein decomposition and fat oxidation of pompano.	(Sun et al., 2022)
Active coating	Pompano(*Trachinotus ovatus*)	CS-g-CA exhibited good antioxidant properties and in vitro antimicrobial activity against *Pseudomonas fluorescens* and *Staphylococcus saprophyticus*. It could effectively delay the deterioration of pompano s' texture.	(Lan et al., 2023)
Active coating	Sea bass	The CA-containing coating effectively inhibited the growth and development of various microorganisms and significantly reduced the growth rates of pH, TVB-N and MDA. The water holding capacity of sea bass was maintained. It could extend the shelf life by 6 days in refrigerated preservation.	(Yang et al., 2022)

### 5.1 Food packaging film application

Chitosan-based films containing CA have shown excellent antimicrobial, antioxidant, and shelf-life-extending properties for fruits (mango and apple). The dense and compact nature of these films reduced water loss. Furthermore, due to the presence of phenolic hydroxyl groups in the CA structure, the films exhibited excellent antimicrobial and antioxidant capacity, thus highlighting the ability to preserve freshness in mango and apple applications. Overall, CA-containing films could be used as antioxidant, water barrier and antibacterial layer in fruit active packaging films, thus effectively preventing water loss and slowing down the ability of fruit to decay (Xu et al., 2022). Mou et al. developed CA-containing chitosan/pullulan polysaccharide-active films (CA-g-CS/Pul, 1:1, *w*/w) and applied to peach preservation. The findings demonstrated that peach spoilage is usually caused by fungi and the CA-g-CS/Pul film exhibited enhanced antifungal activity. This was attributed to the inhibition of 14α-demethylase activity by CA. At the 8th day of storage, peaches from the control group presented extensive skin wrinkling, pulp collapse, browning, and extensive growth of decay-causing fungi. The deterioration of peach quality can be due to internal biochemical reactions resulting from deterioration caused by fungal activity. The control group presented a highly significant rate of decay during storage (*p* < 0.001). This may be due to the fact that peaches preserved without CA-g-CS/Pul film were more susceptible to external damage, as well as fungal infections leading to spoilage. Moreover, the CA-g-CS/Pul film retarded the water loss of peaches during storage and could better maintain the hardness of peaches. During storage, the CA-g-CS/Pul film slowed the rate of increase in malondialdehyde (MDA) content of peaches, suggesting that the active film reduced the damage to cell membrane fluidity and selective permeability caused by high levels of MDA, mitigated oxidative stress, and maintained normal intracellular nutrient exchange. Overall, CA-g-CS/Pul film has a good potential for application in preserving fruits, extending the shelf life of peaches by 2 days (Mou et al., 2025). Chitosan/polylactic acid based active films (CA-g-CS/PLA, 14 %, w/w) containing CA have been applied to preserve Agaricus bisporus mushrooms. Ordinary films (polyethylene, PE) showed significant browning and slight decay from the 9th day, and severe soaking, irregular shapes, and extensive decay on the 15th day. Nevertheless, agaricus bisporus preserved by CA-g-CS/PLA film did not show significant decay even on the 15th day of preservation, with less color difference, less change in hardness, and less change in oxygen and carbon dioxide concentration inside the package. Furthermore, PE-preserved Agaricus bisporus had significantly high respiration rates during storage, which would accelerate nutrient depletion and eventually lead to decay. From the 9th day of preservation, the CO_2_ concentration in the PE group was about 7-fold higher than that in the CA-g-CS/PLA group. This could lead to anaerobic respiration of agaricus bisporus when the carbon dioxide concentration is too high, thus improving browning. Moreover, agaricus bisporus preserved with CA-g-CS/PLA film showed higher CAT activity and lower MDA, O_2_· and H_2_O_2_ contents. Overall, CA-g-CS/PLA film significantly enhanced the storage quality of Agaricus bisporus through multiple regulatory mechanisms, including inhibition of ROS accumulation, enhancement of antioxidant enzyme activities, reduction of MDA content and activation of its own antioxidant defense system (Pei et al., 2022).

**Fig. 4:**
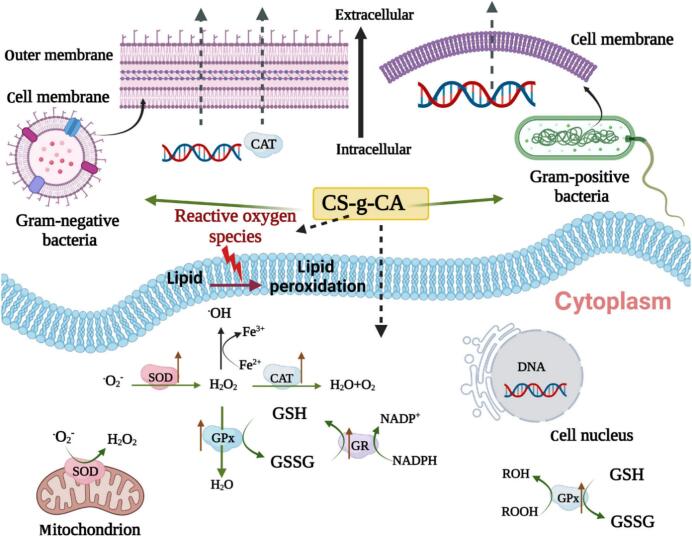
Mechanism of action of antimicrobial activity of CS-g-CA (Lan et al., 2023).

Yong et al. prepared a chitosan (CS)-based active film (CS-g-CA film) containing CA and applied to pork preservation. The results showed that the active film had excellent antioxidant and antimicrobial activities due to CA incorporation **(**Fig. 4**)**. During the pork preservation process, the CS-g-CA film preserved pork exhibited the minimal color difference and the minimal odor. Microbial utilization and degradation of proteins were measured by total volatile base nitrogen (TVB-N). The TVB-N values showed an increasing trend with increasing storage time, while the pork preserved by CS-g-CA films presented the smallest TVB-N values. This indicated that the pork was minimally utilized and degraded by microorganisms, that is, the CA incorporation had an antibacterial and preservation effect. Pork packed in CS-g-CA films exhibited the lowest thiobarbituric acid reactive substances (TBARS) values. This demonstrated that the incorporated CA could trap active oxygen and interrupt the fat oxidation process. Overall, the CS-g-CA film has excellent properties that extend the shelf life of pork at 4 °C up to 10 days (Yong et al., 2021). Polylactic acid (PLA)-based electrospun fiber films containing CA (1 %, *w*/w) have shown excellent barrier properties, antioxidant properties and extended shelf life for pork preservation. During 9 days of storage, the control group showed the fastest decrease in a* (chromaticity value), while the a* of the CA-containing fiber film did not change significantly (*p* > 0.05), indicating that CA could slow down the change in meat color and quality. As storage time increased, microbial degradation of meat proteins and production of amines and ammonia led to an increase in pH. The pH and total volatile base nitrogen (TVB-N) values of pork showed that the pH and TVB-N values of the fiber film containing CA increased significantly less than those of the control (*p* < 0.05). This showed that CA played a role in the preservation of pork. The results of total viable count (TVC) showed that the fiber film containing CA had a lower microbial count, inhibited microbial growth and development, and acted as a preservative after 9 days of storage. In addition, the malondialdehyde (MDA) content showed less MDA after 9 days of storage, which revealed that the presence of CA slowed down the fat oxidation of pork, and these properties are beneficial to the preservation of meat products (Zhu et al., 2025). Lin et al. developed cassava starch-based active films containing CA (1,5, *w*/w) and applied to the preservation of beef and chicken meat. The incorporation of CA resulted in active films with good antioxidant and antimicrobial properties. Compared with the control, the color of meat preserved with the active film showed little change in color after 5 days of storage and slowed down the increase in pH and malondialdehyde values of the meat. These results suggested that CA incorporation reduces the color change of meat and retards lipid oxidation and spoilage of meat. Moreover, during the 5-day storage period, *Escherichia coli* and *Staphylococcus aureus* in the control group showed a rapid growth trend, while the CA-containing active films showed a slow growth trend. This demonstrated that CA could effectively inhibit the growth and development of *Escherichia coli* and *Staphylococcus aureus*, and this bacteriostatic property was also an important reason for the prolongation of shelf life. Furthermore, during the 5-day storage period, the meat products packed with active films showed better elasticity, hardness, stickiness and chewiness (Lin et al., 2023).

### 5.2 Edible coating applications

Osondu et al. developed an active coating containing CA (0.07 %, w/w) based on chitosan hydrochloride (CH-CA) and applied it to the preservation of avocado. The CH-CA coating possessed antifungal activity and was effective in preventing avocado decay caused by anthracnose. This was attributed to its ability to activate defense mechanisms in avocado and may facilitate CA-fungal interactions and cell damage by acting as a favorable adhesion agent between the pathogen and avocado. Sensory experiments demonstrated that CH-CA-coated preserved avocados had better overall acceptability, skin color, texture, flavor, and textural structure. Overall, the coating has the potential to be applied to fruit preservation (Osondu et al., 2022).

Agarose-based active packaging coatings containing CA (1,10, w/w) presented good antioxidant activity with DPPH radical scavenging of about 70 %. Excellent inhibition (100 % inhibition) was observed against *Escherichia coli* and *Staphylococcus aureus*. It may be related to the fact that CA incorporation inhibited bacterial cell wall formation, disrupted bacterial cell membrane permeability and hindered organism metabolism. Grass carp preservation application experiments showed that the active coating containing CA effectively inhibited the growth of histamine-producing bacteria, reduced the rate of fat oxidation, and reduced the production of free fatty acids. Additionally, the CA-containing active coating significantly slowed down the increase in malondialdehyde (MDA) content of grass carp during storage compared with the CA-free coating. This could be attributed to the excellent antioxidant properties of the incorporated CA, which endowed the active coating with antioxidant activity, and the coating was also effective in preventing the grass carp from being exposed to oxygen. In addition, the active coating slowed down the evaporation of water from the fish fillets during the preservation process and the pH range was small during the whole storage period, which suggested that the incorporation of CA can be effective in the preservation of fish and prolong the shelf-life of fish (Fu et al., 2023). Fei et al. developed CA-doped chitosan-based active coatings (Cf-CTS) and applied them to pork preservation. The Cf-CTS coatings exhibited excellent DPPH radical scavenging (77.82 %) and antimicrobial activity (93.39 %). These were attributed to the presence of phenolic hydroxyl groups in the CA structure, which played a role in inhibiting the formation of bacterial cell membranes and disrupting the permeability of bacterial cell walls. After 8 days of storage, the sensory scores (a combination of odor, color, etc.) were significantly higher than those of the coating without CA. The total viable bacteria count of pork preserved with CA-containing coatings was always lower than that of the control group during the storage period, due to the excellent antimicrobial activity of CA. This is also an important reason for the improved shelf life. Fat oxidative rancidity is also another important indicator of the freshness of meat products. The degree of fat oxidation could be reflected by malondialdehyde (MDA) and acid value (AV). The results showed an increasing trend of both MDA and AV with increasing storage time. However, the CA-containing active coating had the lowest MDA and AV, which was attributed to the excellent antioxidant properties of CA, slowing down the oxidation process of pork fat and maximizing the freshness of pork (Fei et al., 2022). Huang et al. developed and applied a chitosan-based active coating (CA@CTS-E) based on CA containing CA for pork preservation. The findings demonstrated that pork spoilage is caused by microbial activity and that determining the degree of spoilage based on the total viable bacteria count is an effective approach. The pure chitosan coating strongly increased the total viable bacterial count on the 11th day of storage, while the CA@CTS-E coating showed no significant increase on the 17th day of storage. This could be explained by the excellent antimicrobial activity of CA. The pH of pork preserved with pure chitosan coating was significantly higher than that of CA@CTS-E coating after the 17th day of storage. This was due to the fact that the proteins of pork were decomposed and utilized by microorganisms to produce nitrogen-containing alkaline volatiles, leading to an increase in the pH of pork. However, the CA@CTS-E coating effectively inhibited the microbial activity, thus reducing the production of nitrogen-containing alkaline substances. Furthermore, CA@CTS-*E*-coated pork had the lowest malondialdehyde (MDA), indicating that the incorporation of CA significantly improved the antioxidant property of the coating and delayed the fat oxidation of pork. It could extend the shelf life of pork by 6 days (Huang et al., 2022). Abdelmalek et al. applied CA -containing polyhydroxybutyrate-based active coatings to sausage preservation. The results showed that the CA-based active coating significantly inhibited microbial growth after 30 days of storage at 4 °C, thus extending the shelf life of the sausages (Abdelmalek et al., 2023).

**Fig. 5:**
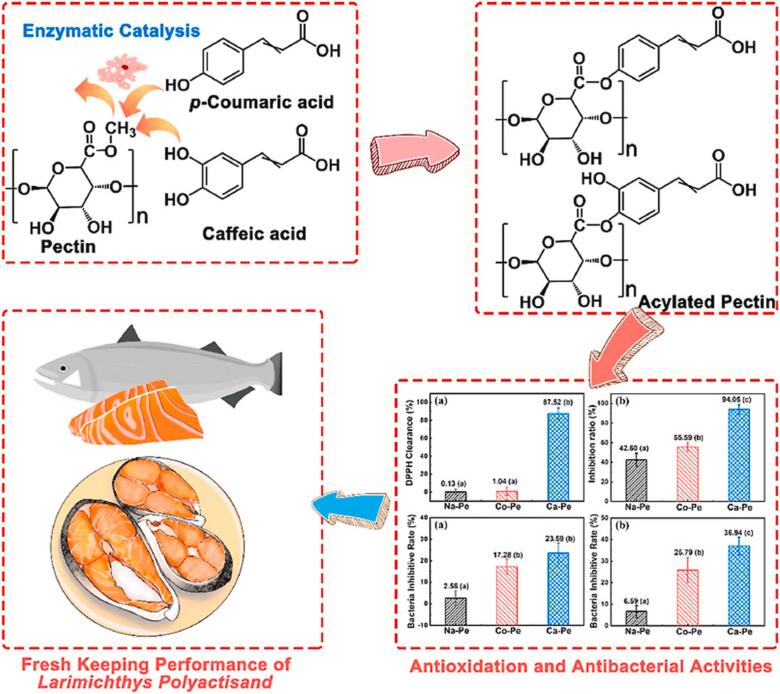
Pectin-grafted caffeic acid prompts antimicrobial and antioxidant enhancement and application to food preservation (Wang, Liu, et al., 2021).

Active packaging coatings containing CA based on acylated pectin exhibited excellent DPPH radical scavenging activity and antimicrobial properties **(**Fig. 5**)**. The results of the freshness preservation experiments on small yellow croaker fillets showed that after 10 days of storage, the CA-containing active coating had the lowest total viable counts (TVCs) and water loss from the small yellow croaker fillets, which was attributed to the bactericidal effect of CA. The total volatile base nitrogen (TVB-N) produced by small yellow croaker fillets during storage was the lowest, indicating that CA incorporation could inhibit microbial growth and production and slow down the food spoilage process. Meanwhile, the degree of oxidation of unsaturated fatty acids in small yellow croaker fillets was also reduced with CA doping. In conclusion, due to the excellent antioxidant and antimicrobial properties of CA, it can effectively inhibit the microbial population, water loss, fat oxidation and protein decomposition during the storage process (Wang, Fei, et al., 2021). Sun et al. prepared a chitosan-based active coating (CS-g-CA) containing CA and successfully applied it to pompano preservation. The results showed that CS-g-CA could effectively inhibit the growth and reproduction of total viable bacteria, *Pseudomonas aeruginosa*, *Cryptophilus aeruginosa*, and H_2_S-producing bacteria. Pompano preserved by the CS-g-CA coating had a better water-holding capacity, indicating that the coating was effective in reducing pompano water evaporation during storage. Furthermore, the CA-free coating showed a significantly higher pH rise during preservation than CS-g-CA. This could be attributed to the antimicrobial effect of CA leading to inhibition of bacterial growth, slowing down the spoilage process of pompano, thereby reducing alkaline amines produced by proteolysis. After 27 days of preservation, the CS-g-CA coating preserved pompano exhibited good hardness and elasticity. This is due to the fact that during storage, microbial action caused the disulfide bonds of muscle proteins to break, hydrophilic and hydrophobic groups to be exposed, and pompano flesh softened and lost its hardness and elasticity, while CA incorporation inhibited this process, thus prolonging the shelf-life. Moreover, CS-g-CA preserved pompano had the lowest total volatile base nitrogen (TVB-N) and malondialdehyde (MDA) compared with normal coating. This indicated that CA incorporation was effective in reducing proteolysis and fat oxidation in pompano (Sun et al., 2022a). Yang et al. developed a chitosan-based active coating containing CA and applied it to the preservation of sea bass. The findings showed that sea bass preserved by the CA-free coating had significantly (*p* < 0.05) increased total viable bacteria counts, H_2_S-producing bacteria and *cryophilic bacteria*. Among them, the high number of *cryophilic bacteria* was an important factor contributing to the deterioration of seafood during the refrigerated preservation stage. The CA-containing coating significantly (*p* < 0.05) reduced the number of *cryophilic bacteria*, thereby extending the shelf life of sea bass by 6 days. The rates of increase in pH, total volatile base nitrogen (TVB-N) and malondialdehyde (MDA) of sea bass preserved by active coatings containing CA were significantly lower than those of ordinary coatings during refrigerated preservation, due to the inhibition of microbial-induced degradation of amino acids by CA, as well as inhibition of volatile alkalinity production. Meanwhile, fat oxidation in sea bass was also inhibited. In addition, the CA-containing active coating enhanced the water-holding properties of sea bass (Yang et al., 2022).

## 6 Challenges and limitations of CA-based films/coatings

Caffeic acid-based active films and coatings face multiple challenges in practical applications: its molecules are susceptible to environmental oxidation, hydrolysis and light, resulting in performance degradation, the need to optimize the formulation and structural modification to enhance the stability; the natural components may lead to an increase in the brittleness of the material, affecting the flexibility and adhesion, and it is difficult to meet the requirements of the mechanical properties of the food packaging, biomedicine and other fields; the high cost of the extraction and composite process, which restricts The cost of extraction and composite process is high, restricting the large-scale production; the additives that may be introduced into the material need to be verified through strict safety to ensure biocompatibility and food contact safety; in addition, there are significant differences in different application scenarios on oxygen barrier, moisture barrier, antibacterial or drug release and other functional requirements, the existing research focuses on the optimization of a single performance, and there is a lack of validation of cross-field adaptability. In the future, we need to break through the bottleneck in stability enhancement, cost control, safety assessment and multi-scenario suitability in order to promote its industrialization.

## 7 Conclusion and outlook

CA has emerged as a transformative agent in biodegradable food packaging, addressing critical limitations of conventional biopolymers through its antioxidant, antimicrobial, and structural reinforcement capabilities. Its integration into films and coatings via direct blending or covalent grafting significantly enhances material performance, enabling applications in preserving perishable foods while aligning with sustainability goals. CA-modified systems also exhibit smart functionalities, such as pH responsiveness and UV protection, positioning them as candidates for next-generation active and intelligent packaging. However, challenges persist in optimizing CA's thermal stability, compatibility with hydrophobic matrices, and uniform dispersion during large-scale production. Future efforts should prioritize eco-friendly grafting techniques (e.g., enzymatic or microwave-assisted methods) to streamline manufacturing and reduce costs. Synergistic combinations of CA with nanomaterials (e.g., metal oxides) could further enhance barrier properties and smart responsiveness, while in-depth safety assessments and regulatory approvals are essential to ensure consumer trust. The transition from lab-scale innovation to commercial viability demands collaboration between academia and industry to address technical and economic barriers. By leveraging CA's multifunctionality, future research can unlock scalable, intelligent packaging systems that minimize food waste and environmental impact, ultimately advancing the global shift toward circular, sustainable food supply chains.

## CRediT authorship contribution statement

**Wanying Sun:** Writing – review & editing, Writing – original draft, Investigation, Data curation, Conceptualization. **Yuxiao Hu:** Writing – original draft, Validation, Formal analysis, Conceptualization. **Ananthi Pandi:** Writing – original draft, Conceptualization. **Guohui Yi:** Writing – review & editing, Writing – original draft, Methodology, Conceptualization. **Zhijian Tan:** Writing – review & editing, Writing – original draft, Supervision, Methodology, Conceptualization.

## Declaration of competing interest

The authors declare that they have no known competing financial interests or personal relationships that could have appeared to influence the work reported in this paper.

## Data Availability

No data was used for the research described in the article.
